# Differentiation between Polymyalgia Rheumatica (PMR) and Elderly-Onset Rheumatoid Arthritis Using 18F-Fluorodeoxyglucose Positron Emission Tomography/Computed Tomography: Is Enthesitis a New Pathological Lesion in PMR?

**DOI:** 10.1371/journal.pone.0158509

**Published:** 2016-07-06

**Authors:** Daisuke Wakura, Takuya Kotani, Tohru Takeuchi, Tsuyoshi Komori, Shuzo Yoshida, Shigeki Makino, Toshiaki Hanafusa

**Affiliations:** 1 Department of Internal Medicine (I), Osaka Medical College, Takatsuki, Osaka, Japan; 2 Department of Radiology, Osaka Medical College, Takatsuki, Osaka, Japan; Nippon Medical School Graduate School of Medicine, JAPAN

## Abstract

**Background:**

It is difficult to differentiate polymyalgia rheumatica (PMR) from elderly-onset rheumatoid arthritis (EORA) in clinical practice. We compared FDG-PET/CT findings between patients with PMR and those with EORA and extracted factors useful for differentiating the two disorders.

**Methods:**

We compared abnormal FDG accumulation sites and maximum standardized uptake value (SUVmax) between 15 patients with PMR and 7 with EORA in whom FDG-PET/CT was performed.

**Results:**

The proportion of patients in the PMR group with abnormal FDG accumulation at the following 9 sites on FDG-PET/CT was significantly higher than that in the EORA group: periarticular region of the scapulohumeral joint, enthesis of the pectineus muscle, vicinity of the enthesis of the rectus femoris muscle, lateral side of the greater trochanter, ischial tuberosity, hip joint, spinous process of the lower cervical vertebra, intervertebral joint of the lumbar vertebra, and spinous process of the lumbar vertebra. The PET/CT score was evaluated at 9 sites consisting of the abovementioned sites. The median score in the PMR group was 8, which was significantly higher than that of 0 in the EORA group (*P* = 0.0003). ROC curve analysis was performed with the PET/CT scores, and a score of 5 was shown to maximize the area under the ROC curve (sensitivity: 86.7%, specificity: 86.7%).

**Conclusions:**

FDG-PET/CT is useful for differentiating PMR from EORA. In patients with PMR, abnormal FDG accumulation was observed at the entheses, suggesting the presence of enthesitis in addition to bursitis and synovitis.

## Introduction

Polymyalgia rheumatica (PMR) is an idiopathic and inflammatory rheumatic disease that develops in elderly persons. It is characterized by morning stiffness and symmetric pains of the neck, shoulder, and pelvis for 1 month or more with concomitant symptoms such as fever, arthralgia, anorexia, weight loss, and depression [1, 2]. The levels of inflammatory biomarkers are high, but autoantibodies such as rheumatoid factor (RF) and anti-cyclic citrullinated peptide (anti-CCP) antibody are negative. Elderly-onset rheumatoid arthritis (EORA) is defined as rheumatoid arthritis that develops at 60 years of age or older. The proportion of patients with involved joints mainly of the proximal limbs and negative autoantibodies is greater than that in younger-onset RA patients [3–5]. Thus, it is difficult to differentiate PMR from EORA in clinical practice.

^18^F-fluorodeoxyglucose positron emission tomography/computed tomography (FDG-PET/CT) facilitates investigation of systemic lesions in a single session. It is useful for detecting malignant diseases and inflammatory lesions such as autoimmune disease. A previous study reported that FDG-PET/CT was useful for detecting angiitis in patients with autoimmune diseases [6]. In PMR, PET or FDG-PET/CT shows abnormal FDG accumulation in the shoulder girdle, pelvic girdle, vertebrae, and sternoclavicular joint [7–9].

In this study, we compared abnormal FDG accumulation at 3 sites (shoulder girdle, pelvic girdle, and perivertebral region) between patients with PMR and those with EORA and examined findings useful for the differential diagnosis of these two diseases.

## Subjects and Methods

### Study design

The subjects included consecutive 15 patients with PMR and 7 with EORA who were admitted to Osaka Medical College Hospital between January 2008 and November 2011 and had undergone FDG-PET/CT. Healey’s diagnostic criteria [2] were used for the diagnosis of PMR. Patients with infection, other collagen vascular diseases, and malignant disease were excluded. The patients with EORA met both the 2010 ACR-EULAR classification criteria for rheumatoid arthritis [10] and the American Rheumatism Association (ARA) 1987 revised criteria [11].

Data on patient background, age, sex, and duration of disease (interval from onset until FDG-PET/CT) were obtained from medical records, as were laboratory data including complete blood cell count, C-reactive protein (CRP) level, erythrocyte sedimentation rate (ESR), matrix metalloproteinase 3 (MMP-3) level, anti-nuclear antibody (ANA), RF, and anti-CCP antibody, and contents of treatment. In the EORA patients, sharp/ van der Heijde score (SHS) were evaluated. This study was approved by the ethics committee of Osaka Medical College (No. 1529) and conducted according to the Declaration of Helsinki. Written informed consent was obtained from each patient.

### FDG-PET/CT imaging

We evaluated sites of abnormal FDG accumulation in the shoulder girdle, pelvic girdle, and perivertebral region of the subjects. For FDG-PET/CT, we used an 8-channel multi-detector computed tomography system (Discovery ST, GE Healthcare, Milwaukee, WI, USA). The patients were fasted for 6 hours or more before the FDG-PET/CT examination. FDG at a dose of 185–370 MBq (5–10 mCi) was intravenously administered 1 hour before imaging. Cross-sectional PET images of the whole body were obtained using a workstation (GE Healthcare). The localization CT was used to interpret uptake location. FDG accumulation on PET/CT is most intense in the brain and urinary bladder, followed by the liver, then the lung and soft tissue, being divided into 3 grades. In our study, regions with stronger FDG accumulation than that in the liver were regarded as basic accumulation-positive. Two specialists in nuclear medicine who were not informed of the subjects’ clinical background or treatment evaluated the FDG-PET/CT images. Accumulation points on FDG-PET/CT were judged based on consensus between 2 radiologists.

### Statistical analysis

Statistical analysis was performed using the Mann-Whitney U-test to compare median values and Fisher’s exact test to compare frequencies. Receiver operating characteristic (ROC) curve analysis was used to determine the most suitable cut-off level. A value of *P* <0.05 indicated statistical significance. The data were analyzed using JMP software, version 11.0 (SAS Institute, Cary, NC, USA).

## Results

### Patient profiles

The patient characteristics are shown in Table 1. The 15 patients with PMR consisted of 5 men and 10 women with a median age of 72 years (range: 55–85). The median duration of disease from onset until PET was 3 months (range: 0.5–22). Five patients were positive for antinuclear antibody. There were no RF- or anti-CCP antibody-positive patients. Giant cell arteritis (GCA) was noted in 1 patient, diagnosed at the same time as PMR diagnosis. In the patient with GCA complication, claudication of the lower limbs, abnormal FGD accumulation in the common iliac over the femoral and popliteal arteries on PET/CT, and hypertrophy of the lower limb arteries on contrast-enhanced MRI were observed. None of the PMR patients met the 2010 ACR-EULAR classification criteria for RA. PMR was improved by median dose 20 mg/day (2.5–35) of prednisolone (PSL) in all of the 15 patients. Three of them were treated with concomitant methotrexate (MTX) because the disease recurred during PSL dose reduction. In the patient with GCA complication, 8 mg/week MTX was concomitantly administered from early treatment. As of December 2015, 14 patients were attending our hospital. The median duration of follow-up was 67 months (59–88), and remission was maintained by PSL 4.25 (0–10) mg/day in 14 patients and MTX 8 mg/day (4–12) in 7. PMR remitted in one patient, but the patient ended their hospital visits by their own will 10 months after treatment initiation. No patient developed RA or was complicated by malignancy throughout the course. Excluding one patient in whom PMR and GCA were simultaneously noted, no patient was complicated by GCA throughout the course.

**Table 1:** Patient characteristics between the PMR and EORA groups.

	PMR (n = 15)	EORA (n = 7)	*P*-value^a^
Age, years	72 (55–85)	77 (60–82)	0.31
Female, n	10	5	1
Disease duration, months	3 (0.5–22)	3.5 (0.5–25)	0.16
SHS	-	162 (85–263)	-
WBC, /μl	8530 (6410–11560)	8230 (4510–14980)	0.42
Neut, %	76.2 (61.1–81.9)	75 (57–85)	0.83
Hb, g/dl	11.2 (7.5–13.6)	11.6 (8.4–12.4)	0.97
PLT, /μl	429000 (268000–76000)	333000 (218000–676000)	0.12
CRP, mg/dl	7.1 (2.8–19.2)	3.4 (0.75–14.1)	0.18
ESR, mm/h	64 (11–120)	61 (23–89)	0.69
MMP-3, ng/ml	347 (110–916)	294 (140–968)	0.81
ANA, n	5	5	0.17
RF-positive, n	0	6	-
Anti-CCPab, n	0	6	-
Angiitis, n	1	0	-

Values indicate the median (interquartile range). PMR: polymyalgia rheumatica; EORA: elderly-onset rheumatoid arthritis; SHS: sharp/ vas der Heijde score; WBC: white blood cell; Neut.: neutrophils; Hb; hemoglobin; PLT: platelets; CRP: C-reactive protein; ESR: erythrocyte sedimentation rate; MMP-3: matrix metalloproteinase 3; ANA: anti-nuclear antibody; RF: rheumatoid arthritis; anti-CCPab: anti-cyclic citrullinated peptide antibody; Angiitis: complication with angiitis.

^a^*P*-values were estimated using Fisher’s exact test or Mann-Whitney U-test.

The 7 patients with EORA included 2 men and 5 women with a median age of 77 years (range: 60–81). Five patients were positive for antinuclear antibody, all 7 patients were positive for RF, and 6 of the 7 patients were positive for anti-CCP antibody. On radiographic examination, the median SHS was 162 (85–263). There were no differences in patient background between the PMR and EORA groups, excluding the proportions of RF- and anti-CCP antibody-positive patients. None of the RA patients met the 2012 provisional ACR/EULAR classification criteria for PMR. To treat EORA, MTX, tacrolimus, and PSL, 3 mg/day were administered to 2, 5, and one patient, respectively. Golimumab, adalimumab, and tocilizmab were each administered to one patient, respectively. One patient transferred to another hospital for treatment of serious dementia 3 weeks after the diagnosis of EORA. RA was in a low disease activity in one patient, but concomitant small cell lung carcinoma developed 7 months after treatment initiation, and the patient transferred to the department of respiratory medicine of another hospital for treatment of the lung cancer. RA remitted in one patient, but the patient transferred to the department of respiratory medicine of another hospital for treatment of status asthmaticus after 6 months. As of December 2015, 4 patients continued treatment at our department, and the disease was in low- and moderately-active states in 2 patients each, respectively. No finding suggesting spondyloarthropathy was noted in any of the PMR or EORA patients.

### FDG-PET/CT findings of PMR

**Fig 1:**
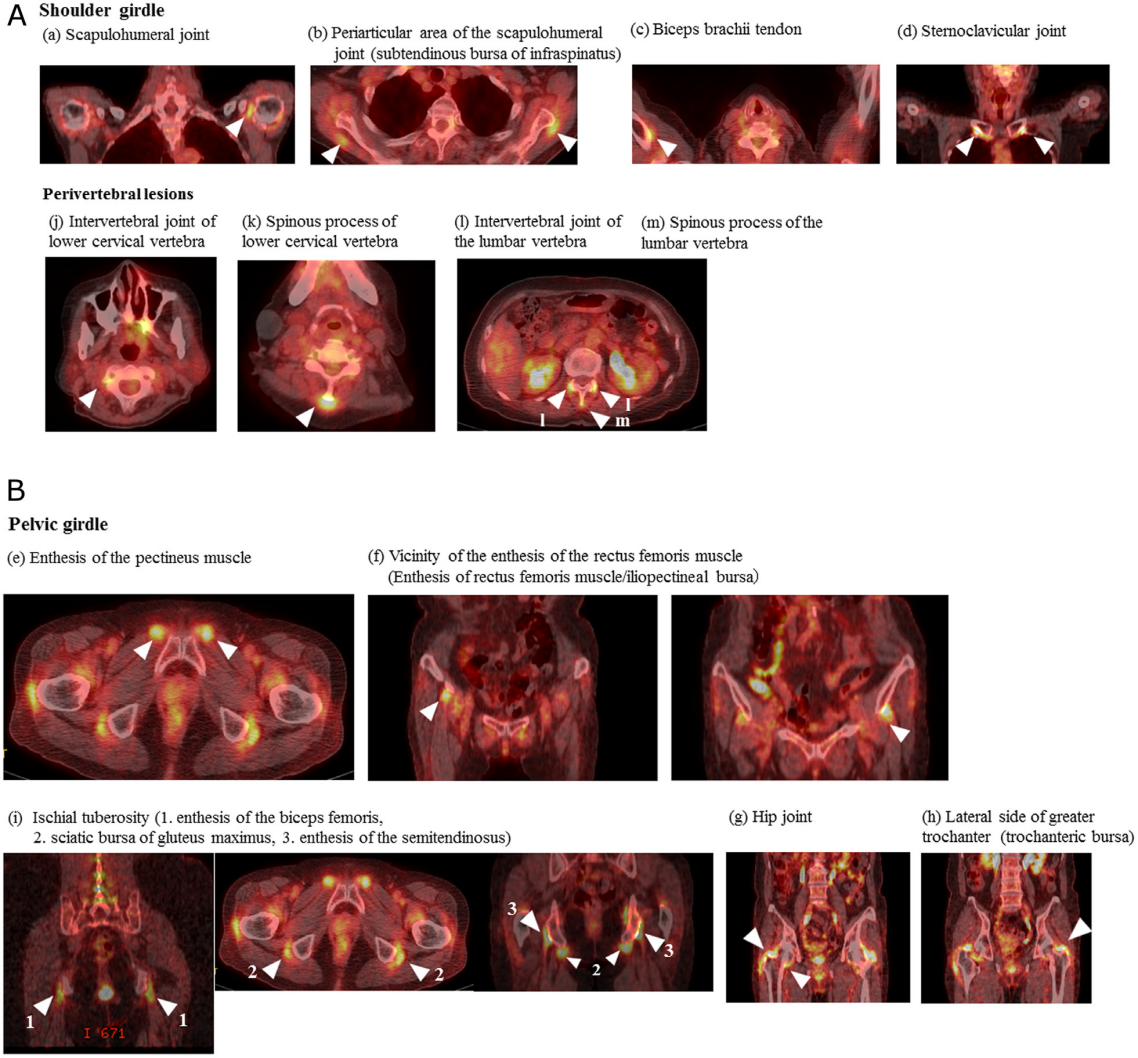
FDG-PET/CT findings of polymyalgia rheumatic. **(A)** Shoulder girdle: (a) scapulohumeral joint (synovitis), (b) periarticular area of the scapulohumeral joint (bursitis, subtendinous bursa of infraspinatus), (c) biceps brachii tendon (vaginal synovitis), and (d) sternoclavicular joint (synovitis). Perivertebral lesions: (j) intervertebral joint of the lower cervical vertebra (synovitis), (k) spinous process of the lower cervical vertebra (bursitis), (l) intervertebral joint of the lumbar vertebra (synovitis), and (m) spinous process of the lumbar vertebra (bursitis). (**B) FDG-PET/CT findings of polymyalgia rheumatica.** Pelvic girdle: (e) enthesis of the pectineus muscle (enthesitis), (f) the vicinity of the enthesis of the rectus femoris muscle (enthesitis, enthesis of the rectus femoris muscle; bursitis, iliopectineal bursa), (g) hip joint (synovitis), (h) lateral side of the greater trochanter (bursitis, trochanteric bursa), and (i) ischial tuberosity (enthesitis and bursitis; 1. enthesis of the biceps femoris, 2. sciatic bursa of the gluteus maximus, 3. enthesis of the semitendinosus).

Representative FDG-PET/CT findings of PMR are shown in Fig 1a and 1b. In the shoulder girdles, abnormal FDG accumulation was observed in the (a) scapulohumeral joint, (b) periarticular area of the scapulohumeral joint, (c) biceps brachii tendon, and (d) sternoclavicular joint. In the pelvic girdles, it was noted in the (e) enthesis of the pectineus muscle, (f) vicinity of the enthesis of the rectus femoris muscle, (g) hip joint, (h) lateral side of the greater trochanter, (i) ischial tuberosity, perivertebral lesion, (j) intervertebral joints of the lower cervical vertebrae, (k) spinous processes of the lower cervical vertebrae, (l) intervertebral joints of the lumbar vertebrae, and (m) spinous processes of the lumbar vertebrae.

### Abnormal FDG accumulation sites on FDG-PET/CT in the PMR and EORA patients

The abnormal sites of FDG accumulation on FDG-PET/CT can be compared between the PMR and EORA patients in Table 2. In the shoulder girdles, the proportion of patients with abnormal accumulation in the periarticular area of the scapulohumeral joint in the PMR group was significantly higher than those in the EORA group (14.3 vs. 73.3%, *P* = 0.02). In the pelvic girdles, the proportion of patients with abnormal accumulation in the following sites in the PMR group was significantly higher than those in the EORA group: the vicinity of the enthesis of the rectus femoris muscle (0 vs. 86.7%, *P* = 0.0002), lateral side of the greater trochanter (42.9 vs. 93.3%, *P* = 0.02), and ischial tuberosity (4.3 vs. 93.3%, *P* = 0.0006). In the PMR group, the proportions of patients with abnormal accumulation in the enthesis of the pectineus muscle and hip joint were significantly higher those in the EORA group (0 vs. 60%, *P* = 0.02; 14.3 vs. 73.3%, *P* = 0.02, respectively). For the perivertebral lesions, the proportions of patients with abnormal accumulation in both the intervertebral joints and the spinous processes of the lumbar vertebrae in the PMR group were significantly higher than those in the EORA group (0 vs. 73.3%, *P* = 0.004; 14.3 vs. 73.3%, *P* = 0.02, respectively). In the former, the proportion of patients with abnormal accumulations in the bilateral intervertebral joints of the lumbar vertebra was significantly higher than that in the latter (0 vs. 73.3%, *P* = 0.004).

**Table 2:** Sites of abnormal FDG accumulation in patients with PMR and EORA.

Abnormal FDG accumulation site		PMR	EORA	*P*-value^a^
Shoulder girdle				
Scapulohumeral joint	Positive, n (%)	12 (80)	4 (57)	0.33
Periarticular area of scapulohumeral joint	Positive, n (%)	11 (73)	1 (14)	0.02*
Biceps brachii tendon	Positive, n (%)	4 (27)	0 (0)	0.26
Sternoclavicular joint	Positive, n (%)	9 (60)	2 (29)	0.36
Pelvic girdle				
Enthesis of pectineus muscle	Positive, n (%)	9 (60)	0 (0)	0.02*
Vicinity of the enthesis of rectus femoris muscle	Positive, n (%)	13 (87)	0 (0)	0.0002*
Hip joint	Positive, n (%)	11 (73)	1 (14)	0.02*
Lateral side of greater trochanter	Positive, n (%)	14 (93)	3 (43)	0.02*
Ischial tuberosity	Positive, n (%)	14 (93)	1 (14)	0.0006*
Perivertebral lesions				
Intervertebral joint of lower cervical vertebra	Positive, n (%)	2 (13)	0 (0)	1
Spinous process of lower cervical vertebra	Positive, n (%)	7 (47)	0 (0)	0.05*
Intervertebral joint of lumbar vertebra	Positive, n (%)	11 (73)	0 (0)	0.004*
Spinous process of lumbar vertebra	Positive, n (%)	11 (73)	1 (14)	0.02*

FDG: fluorodeoxyglucose; PMR: polymyalgia rheumatica; EORA: elderly-onset rheumatoid arthritis.

^a^*P*-values were estimated using Fisher’s exact test.

*P <0.05.

We also compared the maximum standardized uptake value (SUVmax) for abnormal FDG accumulation sites on PET/CT between the PMR and EORA patients and observed no significant differences between the two groups (Table 3).

**Table 3:** Comparison of the SUVmax for abnormal FDG accumulation sites on PET/CT between the PMR and EORA patients.

Abnormal FDG accumulation site	SUVmax		*P* value^b^
	PMR	EORA	
Shoulder girdles			
Scapulohumeral joint	4.5 (2.1–7.3)	4.1 (3.0–7.3)^x^	0.86
Periarticular of scapulohumeral joint	4.5 (2.7–7.6)	3.3^y^	-*
Biceps brachii tendon	3.3 (2.5–4.4)	-	-
Sternoclavicular joint	3.9 (2.4–4.9)	2.9^y^	-*
Pervic girdles			
Enthesis of pectineus muscle	3.5 (2.2–5.9)	-	-
Vicinity of the enthesis of rectus femoris muscle	4.1 (2.2–6.6)	-	-
Hip joint	4.5 (3.2–5.9)	3.1^y^	-*
Lateral side of greater trochanter	3.7 (1.6–6.9)	5.0 (3.6–5.1)^z^	-*
Ischial tuberosity	3.9 (2.1–7.0)	3.5^y^	-*
Perivertebral lesion			
Intervertebral joint of lower cervical vertebra	3.0 (2.5–3.4)	-	-
Spinous process of lower cervical vertebra	3.2 (2.1–4.0)	-	-
Intervertebral joint of lumbar vertebra	3.3 (1–6.4)	-	-
Spinous process of lumbar vertebra	4.5 (2.1–6.6)	1.3^y^	-*

SUVmax: the maximum standardized uptake value; FDG: fluorodeoxyglucose; PMR: polymyalgia rheumatica; EORA: elderly-onset rheumatoid arthritis.

^b^*P*-values were estimated using Mann-Whitney U-test.

*Statistical analyses were not performed because of the small sample number.

^x^n = 4.

^y^n = 1.

^z^n = 3.

### FDG-PET/CT scoring using abnormal accumulation sites

Scoring was performed based on the number of abnormal accumulation points (0–9) at 9 sites: 9 sites at which there was a significant difference in the frequency of abnormal accumulation on FDG-PET/CT between the PMR and EORA groups (periarticular area of the scapulohumeral joint, enthesis of the pectineus muscle, vicinity of the enthesis of the rectus femoris muscle, lateral side of the greater trochanter, ischial tuberosity, hip joint, spinous process of the lower cervical vertebra, intervertebral joints of the lumbar vertebrae, and spinous processes of the lumbar vertebrae). This was termed the PET/CT score.

### PET/CT scores in the PMR and EORA groups

**Fig 2:**
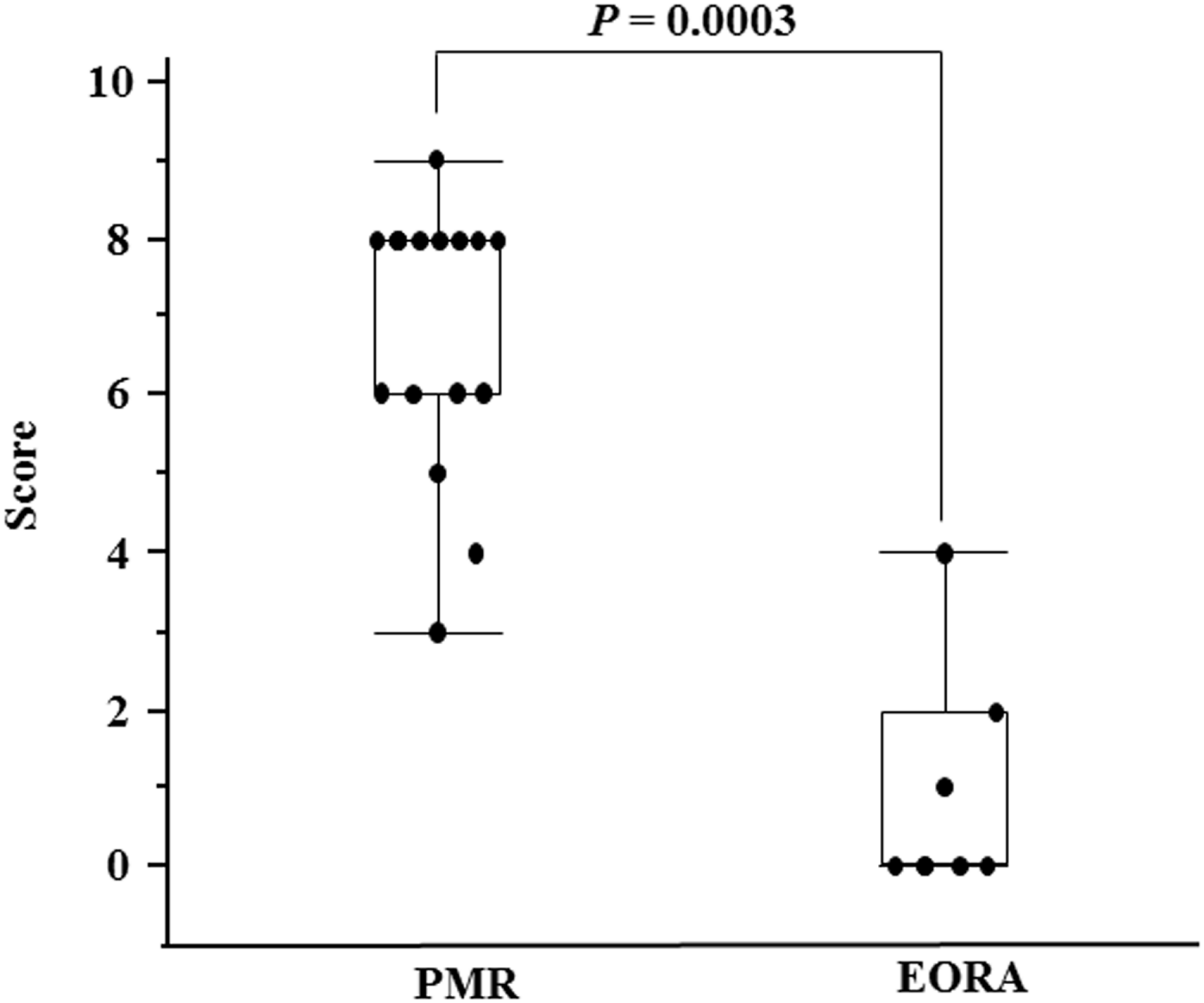
PET/CT scores in patients with PMR and EORA. The PET/CT scores were significantly higher in the patients with polymyalgia rheumatica (PMR) than in the patients with elderly-onset rheumatoid arthritis (EORA) (*P* = 0.0003).

The median PET/CT score in the PMR group was 8 (3–9), whereas that in the EORA group was significantly lower, at 0 (0–4) (*P* = 0.0003) (Fig 2).

### Cut-off value of PET/CT score for differential diagnosis between PMR and EORA patients

To determine cut-off points effective for differential diagnosis between PMR and EORA, ROC curve analysis was performed with the PET/CT scores, and a score of 5 was shown to maximize the area under the ROC curve (sensitivity: 86.7%, specificity: 86.7%). Based on this result, a PET/CT score of ≥5 was determined as the cut-off value for PMR diagnosis.

## Discussion

In this study, we compared FDG-PET/CT findings between patients with PMR and EORA. In the PMR group, the proportions of patients with abnormal FDG accumulation at 8 sites, consisting of the periarticular area of the scapulohumeral joint, enthesis of the pectineus muscle, vicinity of the enthesis of the rectus femoris muscle, lateral side of the greater trochanter, ischial tuberosity, hip joint, intervertebral joints of the lumbar vertebrae, and spinous processes of the lumbar vertebrae, were significantly higher than those in the EORA group. The proportion of patients with abnormal bilateral accumulations at the sternoclavicular joint in the PMR group was significantly higher than that in the EORA group. Scoring was performed at the above 8 sites and bilateral sternoclavicular joints. The PET/CT score of the PMR group was significantly higher than that of the EORA group.

Blockmans et al. examined abnormal accumulation sites of the musculoskeletal system on PET in patients with PMR and found inflammation of the shoulders, hip, and vertebral spinous processes [6]. Camellino and Cimmino reported that FDG-PET/CT findings of PMR included bilateral subacromial/subdeltoid bursitis, peritendinitis of the long head of the biceps brachii muscle, trochanteric bursitis, bursitis of the inter-spinous processes, ischial capsulitis, omarthritis, and sternoclavicular arthritis [9]. Our results support these studies. In addition, we first report here that abnormal accumulation was observed at the attachment sites of the pelvic girdle and intervertebral joints.

Takahashi et al. compared accumulation sites on FDG-PET/CT between 27 patients with PMR and 10 with EORA [7]. In PMR, abnormal accumulation at the ischial tuberosity, vertebral spinous processes, and iliopectineal bursa was significantly higher than that at the wrists and was lower in the shoulders in comparison with EORA. We did not examine accumulation at the wrists, but our findings at the ischial tuberosity, vertebral spinous processes, and iliopectineal bursa were similar. In addition, we observed that the abnormal accumulation at the enthesis of the pectineus muscle, intervertebral joints of the lumbar vertebrae, and bilateral sternoclavicular joints was significantly higher in the PMR group. Thus, these findings of abnormal accumulations of FDG suggest the usefulness of FDG-PET/CT in differentiating the two disorders. However, there was no difference in the SUVmax at abnormal FDG accumulation sites between the two groups.

Bursitis and enthesis may be primarily involved in the pathogenesis of PMR, progressing through synovitis and peripheral inflammation [9, 12, 13]. In addition, the enthesis of the pectineus muscle, the vicinity of the enthesis of the rectus femoris muscle, and the ischial tuberosity are considered to be musculotendinous attachment sites, suggesting the presence of inflammation at the pelvic attachment sites. However, synovitis may be primarily involved in the pathogenesis of RA, progressing through bursitis or peritendinitis [14]. Differences in the primary and secondary lesions between the two disorders may have been reflected by differences in FDG-PET/CT findings.

It is important to differentiate between PMR and EORA at an early stage because of the difference in their prognosis and treatment response. Takahashi et al. scored uptake at the ischial tuberosity, spinous process, and iliopectineal bursa, the absence of uptake at the wrists, and linear or circular uptake around the shoulders on FDG-PET/CT and compared the PET/CT score between PMR and EORA [7]. They reported that this scoring method was useful for differentiating the two disorders. Our results were also similar, but the sites evaluated were different. The sensitivity and specificity of these two scoring systems were high. To advance the PET/CT scoring system, further investigations are needed to show which FDG-PET/CT images are appropriate for the assessment of abnormal accumulation sites.

This was a retrospective study involving a small number of patients. FDG-PET/CT findings were not investigated in seronegative EORA patients and this remains to be investigated in the future. Several studies reported that FDG-PET/CT revealed abnormal accumulation in the articular synovial membrane or attachment area in patients with seronegative spondylarthritis [15, 16]. In the future, differentiation between other rheumatic diseases, including seronegative spondylarthritis, should be further investigated in a larger number of patients, and the PET/CT scoring system needs prospective validation in an independent cohort of patients, preferably in comparison with the another score which was previously reported.

## Conclusions

The results suggest that the FDG-PET/CT findings and scoring system presented here may be useful for evaluating and differentiating PMR from EORA. Although previous reports showed bursitis and synovitis in patients with PMR, we observed abnormal FDG accumulation at the attachment sites of the pelvic girdle in PMR, suggesting the presence of inflammation at these sites.
